# Adapting prescribing criteria for amyloid‐targeted antibodies for adults with Down syndrome

**DOI:** 10.1002/alz.13778

**Published:** 2024-03-13

**Authors:** Hampus Hillerstrom, Richard Fisher, Matthew P. Janicki, Brian Chicoine, Bradley T. Christian, Anna Esbensen, Lucille Esralew, Juan Fortea, Sigan Hartley, Jason Hassenstab, Seth M. Keller, Sharon Krinsky‐McHale, Florence Lai, Johannes Levin, Mary McCarron, Eric McDade, Anne Sophie Rebillat, Herminia Diana Rosas, Wayne Silverman, Andre Strydom, Shahid H. Zaman, Henrik Zetterberg

**Affiliations:** ^1^ LuMind IDSC Foundation Burlington Massachusetts USA; ^2^ Department of Disability and Human Development University of Illinois Chicago Chicago Illinois USA; ^3^ National Task Group on Intellectual Disabilities and Dementia Practices Rockport Maine USA; ^4^ Advocate Health, Advocate Medical Group Adult Down Syndrome Center Advocate Lutheran General Hospital Family Medicine Residency Park Ridge Illinois USA; ^5^ Waisman Center IDDRC University of Wisconsin Madison Wisconsin USA; ^6^ Division of Developmental and Behavioral Pediatrics Cincinnati Children's Hospital Medical Center University of Cincinnati College of Medicine Cincinnati Children's Hospital Cincinnati Ohio USA; ^7^ California Department of Developmental Services Sacramento California USA; ^8^ Biomedical Research Institute Sant Pau Universitat Autònoma de Barcelona Barcelona Spain; ^9^ Department of Neurology Hospital de la Santa Creu i Sant Pau Barcelona Spain; ^10^ Departments of Neurology and Psychological & Brain Sciences Knight Alzheimer Disease Research Center Washington University St. Louis Missouri USA; ^11^ Neurology Associates of South Jersey Lumberton New Jersey USA; ^12^ Department of Psychology New York State Institute for Basic Research in Developmental Disabilities Island New York USA; ^13^ MGH Neurology Research Mass General Brigham Hospital Massachusetts General Hospital Boston Massachusetts USA; ^14^ Department of Neurology & German Center of Neurodegenerative Diseases (DZNE) e.V. Ludwig‐Maximilians University Munich Germany; ^15^ Department of Neurology Sahgrenska University Hospital Mölndal Sweden; ^16^ Trinity Centre for Ageing and Intellectual Disability Trinity College, University of Dublin Dublin Ireland; ^17^ Outpatient Department Institut Jerome Lejeune Paris France; ^18^ Department of Radiology Athinoula Martinos Center Massachusetts General Hospital Harvard Medical School Charlestown Massachusetts USA; ^19^ Department of Pediatrics University of California Irvine Irvine California USA; ^20^ Institute of Psychiatry Psychology and Neuroscience, Kings College London UK; ^21^ Department of Psychiatry Cambridge Intellectual and Developmental Disabilities Research Group Cambridge University Cambridge UK; ^22^ Institute for Stroke and Dementia Research Sahlgrenska Academy at the University of Gothenburg Molndal Sweden; ^23^ Clinical Neurochemistry Laboratory Sahlgrenska University Hospital Molndal Sweden; ^24^ Munich Cluster for Systems Neurology (SyNergy) Munich Germany

**Keywords:** Alzheimer's disease, anti‐amyloid immunotherapeutics, dementia, Down syndrome, drug formularies, prescribing criteria

## Abstract

Prior authorization criteria for Federal Drug Administration (FDA) approved immunotherapeutics, among the class of anti‐amyloid monoclonal antibodies (mAbs), established by state drug formulary committees, are tailored for adults with late‐onset Alzheimer's disease. This overlooks adults with Down syndrome (DS), who often experience dementia at a younger age and with different diagnostic assessment outcomes. This exclusion may deny DS adults access to potential disease‐modifying treatments. To address this issue, an international expert panel convened to establish adaptations of prescribing criteria suitable for DS patients and parameters for access to Centers for Medicare & Medicaid Services (CMS) registries. The panel proposed mitigating disparities by modifying CMS and payer criteria to account for younger onset age, using alternative language and assessment instruments validated for cognitive decline in the DS population. The panel also recommended enhancing prescribing clinicians' diagnostic capabilities for DS and initiated awareness‐raising activities within healthcare organizations. These efforts facilitated discussions with federal officials, aimed at achieving equity in access to anti‐amyloid immunotherapeutics, with implications for national authorities worldwide evaluating these and other new disease‐modifying therapeutics for Alzheimer's disease.

## 1 INTRODUCTION

Adults with Down syndrome (DS) have a genetic predisposition to early onset brain amyloid deposition and face a cumulative lifetime risk of approximately 90% for DS‐associated Alzheimer's disease (DS‐AD). Risk for exhibiting clinical symptoms of DS‐AD increases prior to age 60,^1^, ^2^ contributing to 70% to 80% of deaths in adults with DS.^3^, ^4^ Adults with DS are the largest population with an increased risk for Alzheimer's disease (AD) associated with a specific genotype, as an extra copy of the gene coding for amyloid precursor protein (APP) located on chromosome 21 leads to its overproduction and consequent atypical deposition of amyloid beta (Aβ).^5^, ^6^ Adults with DS typically show Aβ plaque buildup by their fourth decade of life, initiating the cascade of AD neuropathology some 15 to 20 years earlier than is typical for sporadic or late‐onset AD (LOAD).^2^, ^7^, ^8^ Therefore, there is a compelling case for this high‐risk population to have equitable and timely access to new anti‐amyloid monoclonal antibodies (mAbs), which are considered disease‐modifying therapies (DMTs) that slow AD progression.^9^, ^10^

Current prior authorization criteria for the use of anti‐amyloid mAbs, as established by drug formulary committees in the United States, have focused on LOAD in its early stages,^11^, ^12^ and therefore, some of the recommended criteria for prescribing these agents would effectively bar adults with DS‐AD from having access to these innovative treatments. To address this issue, it is necessary to adapt the current inclusionary prior authorization criteria as follows: (1) modify the current age criteria in recognition of average onset of DS‐AD at a younger age compared to LOAD; (2) remove exclusions for adults with pre‐existing lifelong cognitive impairments; (3) allow for diagnostic findings based on neurocognitive and behavioral assessments validated for measuring cognitive and functional decline for adults with DS and other comparable developmental disorders; and (4) address inappropriate contraindications for co‐occurring conditions in adults with DS‐AD, if safety is not affected.

Drug formulary committees have the primary objective of overseeing and designating preferred drugs to guide rational prescribing practices.^13^ State drug formulary committees have assumed the responsibility of selecting precise wording and criteria for Leqembi and any other anti‐amyloid antibodies that gain approval, derived from language from the Food and Drug Administration (FDA) approval label that defines clinical trial patient inclusion criteria. These state committees determine the eligibility for anti‐amyloid mAbs treatment through so‐called prior authorization prescribing criteria, which vary among individual states in defining the eligible LOAD population. These criteria consider factors such as age, exclusion of non‐Alzheimer's dementia, demonstrated cognitive decline or impairment caused by mild cognitive impairment or mild AD, and biomarker indicators for the presence of amyloid plaques (see Appendix C in Hillerstrom et al. ^14^). Additionally, there are variations among states in the applications and provisions for use, only some of which allow for the inclusion and assessment of individuals with a history of intellectual disability (ID), including adults with DS. In fact, some state prior authorization prescribing criteria specifically exclude individuals with DS due to their pre‐existing cognitive impairments (see Appendix C in Hillerstrom et al.^14^).

Most state drug formulary committees’ prescribing criteria reference specific assessments for identifying cognitive impairment in the sporadic AD population. However, these assessments are not suitable for quantifying cognitive decline in the presence of pre‐existing intellectual impairments, highlighting the need for alternative methods specifically tailored for adults with DS.^12^ The typical process for assessing adults with DS and other people with ID entails gathering information from both adults with DS or ID and their caregivers regarding physical and mental health, determining overall adaptive functioning, and utilizing a range of directly administered neuropsychological tests that assess memory and other domains of cognition and signs of cognitive decline.^15^ Furthermore, state prescribing guides direct physicians to adhere to protocols for conducting comprehensive clinical workups and medication reviews to identify and rule out alternative or treatable causes for cognitive decline. Since many adults with DS may have co‐occurring conditions that have been present since childhood,^16^ monitoring these conditions for any potential adverse effects on behavior and/or function is crucial.

Obtaining magnetic resonance imaging (MRI) and positron emission tomography (PET) scans or collection of cerebral spinal fluid (CSF) to meet brain imaging and fluid biomarker AD diagnostic criteria, respectively, in adults with DS can be challenging under the best of circumstances, but it is feasible for specialized clinics or by clinicians highly familiar with this population and depending on the patient's level of tolerance. Given the extremely high likelihood of early‐age amyloid neuropathology in adults with DS,^1^, ^7^ consideration should be given to empirically supported alternatives that can be more easily obtained, such as validated blood biomarkers.^17^, ^18^

Given the high risk for AD‐mediated early‐onset dementia in adults with DS and the potential therapeutic value of anti‐amyloid DMTs, the Working Group on Criteria for Access to Alzheimer's Therapeutics for Adults with Down Syndrome (“Working Group”) determined that there was an urgent need to adapt and enact reasonable accommodations to the current prescribing criteria, while at the same time ensuring that safety risks are managed, consistent with best clinical practice guidelines. While similar concerns have been raised for sporadic AD,^19^ this effort is focused on adaptations specific to DS. Thus, the Working Group comprised a set of evidence‐based and evidence‐informed recommendations for modifying wording in prior authorization prescribing criteria to address this inequity. This report, thus, is intended to (1) propose alternative inclusionary wording and suggested accommodations, and (2) serve as a roadmap or guide for prescribers when determining eligibility for adults with DS.

## 2 WORKING GROUP PROCESS TO GENERATE THE ADVISORY

Under the auspices of the LuMind IDSC Foundation and the National Task Group on Intellectual Disabilities and Dementia Practices, a multinational working group of experts specializing in the clinical, biomarker, and cognitive and behavioral assessment aspects of DS‐AD was convened in February 2023. The participants represented diverse perspectives and expertise related to prescribing criteria for assessing adults with DS‐AD. Their objective was to examine the existing prescribing criteria utilized in various US states for the sporadic AD population and to determine which criteria were applicable to adults with DS‐AD and which required additional wording or modifications. The panel also took into consideration the criteria employed by the US Department of Veterans Affairs and the appropriate use criteria for Leqembi.^20^ At the time, the FDA had given conditional approval for Aduhelm and Leqembi; subsequently, in July 2023, the FDA gave Leqembi traditional (full) approval.^1^ The group's work comprised four phases: defining parameters, reviewing existing criteria, collating perspectives and comments, and reaching a consensus on recommendations.

### 2.1 Derivative data

A search for available state drug formulary information resulted in the compilation of 12 states’ criteria, considered a representative sample, based upon geography, content, and breadth of wording. The criteria of the Veterans Affairs National Formulary were also included as the US Department of Veterans Affairs maintains its own prescribing and insuring processes.^20^ Lastly, the working group considered use and contraindication criteria in the Appropriate Use Criteria published for Aduhelm and for Leqembi.^11^, ^12^ The working group reviewed the criteria based on aspect focus, language, breadth of specification, and presence of formal exclusionary language related to DS (see Appendix C in Hillerstrom et al.^14^).

### 2.2 Consensus

Comments and recommendations for adaptations were discussed during several virtual meetings and via written commentary over the course of approximately 90 days. It culminated with the issuance of an extensive report containing an expanded advisory and consensus statement.^14^

### 2.3 Advisory outcomes

#### 2.3.1 Current criteria

The Working Group conducted an examination of the criteria from 12 representative state drug formulary committees to assess their content and applicability. The categories typically encompassed in the prescribing use criteria include (1) age, (2) prescriber qualifications, (3) validated assessment scales for early AD diagnosis, (4) biomarkers indicating amyloid positivity, (5) evidence of progressive cognitive impairment based on testing, (6) baseline MRI requirements, and (7) exclusion of non‐Alzheimer's causes for cognitive decline. Some states opted for a concise approach, presenting criteria in a terse, straightforward manner, while others provided expanded descriptors for improved clinical precision and measures. Consistencies were found across many of the criteria, but variations and omissions were also noted, pointing to a need for discussions leading to a national consensus, perhaps spearheaded by an appropriate federal agency.

#### 2.3.2 Wording adaptations

The Working Group concluded that the prior authorization prescribing criteria, as defined by states, should be adapted or supplemented to enable the inclusion of adults with DS‐AD. These included seeking, when feasible, guidance from clinical consultants with expertise in diagnosing and managing dementia in adults with DS (or comparable developmental disorders). The Working Group produced the recommended wording (as shown in Tables 1 and 2) for use by state drug formulary committees when modifying or supplementing criteria to accommodate prescribing for adults with DS‐AD (or LOAD‐affected adults with histories of other developmental disorders). As many states have suggested the age of 50 as the floor for drug recipients, the Working Group also recommended either removing the floor age or providing exceptions for DS‐AD as some adults are diagnosed with early AD in their 40s.^21^

**TABLE 1:** Recommended language modifications or additions to state prescribing criteria for Alzheimer's disease treatment medications for adults with Down syndrome.

Criteria	Recommendations and commentary
**State authorization criteria**	
**Age**	**Recommendation**: Patient with Down syndrome may be 50 to 85 years old – or younger and meets other criteria for early DS‐AD.
**Prescriber**	**Recommendation**: For patients with Down syndrome, prescriber should consult with specialist health provider/clinician knowledgeable in DS‐AD or in dementia with intellectual disability, if feasible.
**Validated MCI/mild AD diagnosis assessment scales**	**Recommendation**: For patients with DS, provider attestation for diagnosis of early DS‐AD via evidence of cognitive, functional, and behavioral decline from DS‐appropriate assessments and/or caregiver/informant/clinician interview reports.
**Biomarkers for amyloid positivity**	**Recommendation**: For patients with DS, PET scan is positive for amyloid beta plaque indicative of AD.
**Test evidence of cognitive impairment**	**Recommendation**: For patients with Down syndrome, evidence of cognitive decline relative to premorbid cognitive functioning level, as evidenced by informant‐reported and directly administered assessment measures showing poorer than expected performance.
**MRI at baseline**	**Recommendation**: For patients with Down syndrome, baseline brain MRI scan to assess amyloid‐related imaging abnormalities (ARIA) prior to initiating treatment (within 1 year prior).
**Exclusion of other causes of cognitive impairment**	**Recommendation**: Patients with Down syndrome (DS) are not to be excluded based on lifelong DS‐associated pre‐existing cognitive impairment.

Abbreviations: AD, Alzheimer's disease; DS, Down syndrome; DS‐AD, Down syndrome‐associated Alzheimer's disease; MCI, mild cognitive impairment; MRI, magnetic resonance imaging; PET, positron emission tomography.

**TABLE 2:** Recommended language modifications or additions to other criteria related from Department of Veterans Affairs authorization or to Leqembi appropriate use criteria for adults with Down syndrome.

Criteria	Recommendations and commentary
**Thyroid levels**	**Recommendation**: For patients with DS, hypothyroidism is diagnosed and treated according to standard of care with thyroid stimulating hormone (TSH) levels monitored.
The following are taken from additional Leqembi appropriate use criteria	
**BMI**	**Recommendation**: No significant difference in DS.
**Care partner**	**Recommendation**: No significant difference in DS.
**Understand requirements for therapy**	**Recommendation**: No significant difference in DS.
**Recent history of stroke, transient ischemic attacks, and seizures**	**Recommendation**: For patients with DS, no significant difference of criteria for stroke or transient ischemic attacks; however, as a history of seizures is more likely for individuals with DS and adult‐onset seizures can occur with AD progression, their presence should not be a contraindication for treatment with immunotherapies.
**Mental issues**	**Recommendation**: For patients with DS, mental health criteria are not appropriate as contraindication for immunotherapy treatment, as severe mental illness comorbidities are uncommon.
**Depression**	**Recommendation**: No significant difference in DS.
**Bleeding disorder**	**Recommendation**: No significant difference in DS.
**Anticoagulants**	**Recommendation**: No significant difference in DS.
**Immunological disease**	**Recommendation**: For patients with DS, rheumatoid arthritis, celiac disease, and alopecia areata or totalis, should not be exclusionary in DS‐AD when these conditions are stable. Otherwise, no significant difference in DS for the other immunological diseases referred to in the Appropriate Use Criteria.
**Medications**	**Recommendation**: No significant difference in DS.

Abbreviations: AD, Alzheimer's disease; DS, Down syndrome; DS‐AD, Down syndrome‐associated Alzheimer's disease; DVA, US Department of Veterans Affairs.

## 3 COMMENTARY

The Working Group recommends that the prior authorization prescribing criteria established by states for equivalency be adjusted or supplemented to promote equity by granting access to the emerging class of amyloid targeting AD therapies for adults with DS‐AD and preventing exclusion of adults with DS. The Working Group recognizes concerns related to safety and to better understanding the risks and benefits of the anti‐amyloid DMTs for adults with DS, but to serve its stated primary objective, it limited its focus to drug access equity. The need for specific attention to and adaptations for inclusion and equity for adults with DS‐AD is supported by two key factors: (1) the well‐documented high lifetime risk and early onset of DS‐AD compared to LOAD and (2) the potential efficacy of new mAbs for reducing Aβ accumulation during middle age and the possibility of enhancing both lifespan and brain health in later years. It is not expected that all adults with DS/ID will qualify using clinical trial eligibility criteria for the DMTs, analogous to the LOAD population for which only a small proportion of potentially eligible adults qualified recently due to exclusions.^22^ Such exclusions include some related to other chronic conditions and neuroimaging findings or due to an inability to tolerate essential procedures such as blood draws, injections, or MRI to monitor for the development of potential complications.^22^ On a broader scale, the Working Group recommendations may also have relevance to the work of the Alzheimer's Association and National Institute for Aging's revised diagnostic criteria for a biological definition of AD.^23^, ^24^

The Working Group's efforts identified critical needs to adapt and supplement state‐established prior authorization prescribing criteria, stemming from clinical trial eligibility, to ensure fair access to treatment for adults with DS‐AD. These recommended prescribing adjustments are aimed at not only promoting inclusion and equity but also preventing the exclusion of individuals with DS (or other developmental disorders) from accessing emerging AD therapies. The Working Group's primary focus was on ensuring access equity, although they acknowledged concerns related to the safety and efficacy of DMTs for adults with DS‐AD.^25^

One significant challenge highlighted by the Working Group is the scarcity of experts specializing in DS‐AD in the United States, making it difficult to find clinical consultants. To bridge this gap, the group recommend two key actions:

1. Governmental entities and academic health sciences/medical institutions should establish resources easily and broadly accessible to prospective prescribers, providing them with sufficient information needed to inform their diagnostic and treatment decisions. These resources could be integrated with existing national, state, and other non‐profit organizations focused on public health.
2. Non‐profit organizations and professional/interdisciplinary associations associated with DS/ID and the geriatric medicine community should develop technical resources (such as practice guidelines and help lines). These resources could serve as alternatives to consulting clinical experts related to assessing assessment scale instruments or conducting clinical evaluations, particularly in regions with limited consultants.

Additionally, given the vagaries of prescribing behaviors and resources within the general population,^26^ there is a need to organize continuing education programs tailored specifically for cognitive assessment of adults with DS (eg, via ECHO, telehealth, webinars) and provide for the distribution of guidelines for assessing adults with DS and cognitive decline or recognizable early dementia (eg, Moran et al.^27^; Tsou et al.^28^). See Hillerstrom et al.^14^ on activities that could be undertaken.

Mattke et al.^29^ noted the general difficulties in undertaking brief cognitive assessments (BCAs) in adults. However, even if clinicians can effectively undertake such BCAs with adults with LOAD, they must also be alert to the unique characteristics of DS‐AD and DS cognitive phenotypes. Further, prescribing clinicians need to be able to differentiate between DS‐AD‐related decline and lifelong cognitive limitations. While a small minority of adults with DS may have cognitive capabilities aligning closely with average intellectual capacities, the majority have lifelong impairments, possibly severe, complicating recognition of changes that accompany the transition from the preclinical to prodromal stage of DS‐AD. Informant‐reported brief screening measures, commonly used in this population, can help caregivers evaluate cognition, daily functioning, and behavior, but the need for easily administered, empirically supported direct tests with high sensitivity and specificity persists. The Working Group acknowledged that methods used in research focused on DS‐AD, while highly effective, would need to be streamlined for use in most clinical settings and that longitudinal studies are ongoing that may validate methods that can be more easily completed during routine patient visits.^30^

A pressing concern is the potential delay in adapting prior authorization prescribing criteria for adults with DS‐AD. This delays treatment access to anti‐amyloid mAb therapeutics that have already received FDA approval and to future treatment innovations, as well as extending lack of recognition to be enrolled in clinical trials.^31^ The upside of such a delay is time gained for gathering more treatment experience and for addressing safety issues arising from the general LOAD population^32^ and for new DMTs, such as a third anti‐amyloid mAb, donanemab,^33^ pending approval.^34^ The Working Group also acknowledges the potential for added risks of anti‐amyloid mAb‐induced adverse events, compared to the LOAD population, arising from brain edema and microhemorrhage due to higher rates of cerebral amyloid angiopathy in adults with DS.^35^ To address this additional risk factor, the Working Group's prescribing recommendations presuppose the establishment of safety with anti‐amyloid mAb treatments through clinical study in the DS‐AD population, as also implied by current recommended use guidelines for approved anti‐amyloid mAbs.^11^, ^12^

When the Centers for Medicare & Medicaid Services (CMS) announced its insurance coverage commitment in July 2023 for patients prescribed fully approved anti‐amyloid mAbs for early AD,^36^ provided these patients entered an approved registry, it created an opportunity to engage in critical discussions with CMS officials. These conversations aimed to secure accommodations within the CMS‐approved registries for adults with DS and to verify eligibility for coverage under Medicare and Medicaid. As previously noted,^19^ the current registry structure has limits, as it may have insufficient robustness to permit “a comprehensive analysis of the benefits and harms of drugs in the diverse population of Medicare beneficiaries with comorbidities, disabilities, and who are from demographic categories not adequately represented in clinical trials.” Further, the authors recognized that there are “multiple cognitive, function, and amyloid test options, and every effort should be made to standardize outcome data.” Thus, our effort has been directed at adding a DS‐AD pull‐down option for dementia type and introducing standardization in the test options relevant to adults with DS. This is the direct result of the Working Group's translation of recommendations into practical solutions that would produce equitable access for individuals with DS‐AD to essential therapies after safety issues are addressed, as described above for anti‐amyloid mAbs.

Considering the acknowledgement from CMS of coverage for AD DMTs for patients eligible for Medicare/Medicaid, we propose that state drug formulary committees should revise their prescribing language to be more inclusive and that CMS‐approved registries incorporate applicable instruments for use with patients with DS‐AD. CMS has acknowledged that current coverage policy does not prohibit adults with DS‐AD from accessing approved DMTs.^37^ Consequently, as some adults with DS‐AD will seek access to approved DMTs and be entered into CMS‐approved registries prior to a complete understanding of the safety and efficacy for this specific population,^25^, ^37^ registries should include in their collected data the impact of each specific drug on cognitive improvement and adverse events.^19^ Upon request from CMS, the Working Group is currently engaged in recommending DS‐specific instruments as equivalents for the CMS approved registries.

Overall, the efforts of the Working Group led to extensive outreach to various stakeholders on the imperative of achieving equitable access to new AD therapies, coupled with the necessary language modifications to move that equity toward a reality. This multifaceted outreach strategy encompassed a series of informational sessions and meetings that brought together an array of influential entities and stakeholders. These included the federal Advisory Council on Alzheimer's Research, Care, and Supports, which is responsible for developing the *National Plan to Address Alzheimer's Disease*,^38^ and an “in conjunction with” meeting held at the 2023 Conference of the American Academy of Neurology.^39^ The engagement also extended to prominent federal agencies such as the National Institutes for Health, the National Institute on Aging, the Administration on Community Living, FDA, and CMS, fostering a collaborative environment to align policies and practices with the project's goals. Simultaneously, efforts were undertaken with stakeholder organizations, such as the National Association of Medicaid Directors, the National Association of State Directors of Developmental Disability Services, the Alzheimer's Association, the LEAD Coalition, and others, to amplify the reach and impact of the advocacy efforts.

Additionally, the process undertaken by the Working Group to address language and criteria modifications in the United States may offer valuable insights for other countries as jurisdictions approve anti‐amyloid mAbs use.^40^, ^41^, ^42^, ^43^ Since drug approval and prescribing criteria decisions often occur at the national level, the Working Group's experience can serve as a model for advocating for equity in access within other countries,^44^ especially through advocacy by intellectual disability or DS advocacy organizations.^45^ Its recommendations and insights can also serve as a valuable resource for policymakers, healthcare providers, and advocates working toward improving the quality of care and life for this at‐risk population.

## CONFLICT OF INTEREST STATEMENT

HZ has served on scientific advisory boards and/or as a consultant for Abbvie, Acumen, Alector, Alzinova, ALZPath, Annexon, Apellis, Artery Therapeutics, AZTherapies, Cognito Therapeutics, CogRx, Denali, Eisai, Nervgen, Novo Nordisk, Optoceutics, Passage Bio, Pinteon Therapeutics, Prothena, Red Abbey Labs, reMYND, Roche, Samumed, Siemens Healthineers, Triplet Therapeutics, and Wave, has given lectures at symposia sponsored by Alzecure, Biogen, Cellectricon, Fujirebio, Lilly, and Roche, and is a co‐founder of Brain Biomarker Solutions in Gothenburg AB (BBS), which is a part of the GU Ventures Incubator Program (outside submitted work). The other authors report not having any conflicts of interest. Author disclosures are available in the supporting information.

## Supporting information

Supporting Information

## Footnotes

1 In January 2024, Biogen withdrew support for Aduhelm and terminated any ongoing clinical trials.
